# End Group Stability of Atom Transfer Radical Polymerization (ATRP)-Synthesized Poly(*N*-isopropylacrylamide): Perspectives for Diblock Copolymer Synthesis

**DOI:** 10.3390/polym11040678

**Published:** 2019-04-13

**Authors:** Artjom Herberg, Xiaoqian Yu, Dirk Kuckling

**Affiliations:** Department Chemie, Universität Paderborn, Warburger Str. 100, D-33098 Paderborn, Germany; artjom.herberg@uni-paderborn.de (A.H.)

**Keywords:** controlled radical polymerization, atom transfer radical polymerization, end group determination, *N*-isopropylacrylamide, block copolymerization, smart polymers, temperature sensitive polymers, lower critical solution temperature, ESI-TOF mass spectrometry, ion mobility separation, size exclusion chromatography

## Abstract

Studies on the end group stability of poly(*N*-isopropylacrylamide) during the atom transfer radical polymerization (ATRP) process are presented. Polymerization of *N*-isopropylacrylamide was conducted in different solvents using a copper(I) chloride/Me_6_Tren catalyst complex. The influence of the ATRP solvent as well as the polymer purification process on the end group stability was investigated. For the first time, mass spectrometry results clearly underline the loss of ω end groups via an intramolecular cyclization reaction. Furthermore, an ATRP system based on a copper(I) bromide/Me_6_Tren catalyst complex was introduced, that showed not only good control over the polymerization process, but also provided the opportunity of block copolymerization of *N*-isopropylacrylamide with acrylates and other *N*-substituted acrylamides. The polymers were characterized using ^1^H-NMR spectroscopy and size exclusion chromatography. Polymer end groups were determined via ESI-TOF mass spectrometry enhanced by ion mobility separation (IMS).

## 1. Introduction

The development of controlled radical polymerization (CRP) techniques in the early 1990s enabled the synthesis of polymers with advanced architecture [1,2,3,4]. An efficient and rapid initiation step as well as minimized radical termination are the basic characteristics of CRP. A fast equilibrium between a dormant and an active species leads to a consistent growth of all polymer chains, thus providing low polydispersities (PDIs) and tunable chain lengths [5]. Control over end group functionality is not only an additional key feature of CRP but also an inevitable prerequisite for the synthesis of polymers with advanced architecture. Block copolymer synthesis, for instance, can be achieved by sequential monomer addition or by the use of macroinitiators. In the latter case, the two distinct blocks can be synthesized by a combination of two different polymerization techniques, such as CRP and ring-opening polymerization (ROP) [6,7]. Sometimes, orthogonality allows the simultaneous synthesis of both blocks starting from a bifunctional initiator [8,9]. Even direct coupling of two separately formed polymer blocks via efficient click chemistry can afford block copolymers [10].

Atom transfer radical polymerization (ATRP) is one of the major CRP techniques that is widely used to prepare polymers with predictable chain lengths, narrow molecular mass distributions, predefined compositions, and different functionalities [11,12]. Even complex polymer architectures, such as polymer brushes and star polymers, could be synthesized using this method [13,14,15]. The ATRP is based on a redox equilibrium between the dormant and the active species, catalyzed by a transition metal complex. Thus, the propagating polymer radical is reversibly deactivated by transfer of a halide atom from the transition metal complex. During this step the transition metal is reduced. Because of its storage stability as well as commercial availability, copper represents the most common ATRP catalyst, usually complexed by *N*-donor ligands. As a consequence of the halide atom transfer, the ω-chain end is terminated by an alkyl halide. In literature there are numerous examples describing the end group characterization and modification for poly(acrylates) and poly(styrenes) synthesized by ATRP [16].

*N*-substituted acrylamides are of particular interest as building blocks for polymers, since the nature of substituents has a tremendous impact on the hydrophilic/lipophilic balance of the resulting macromolecule. Thus, several *N*-substituted acrylamides, for instance *N*-isopropylacrylamide (NIPAAm), are known to exhibit a lower critical solution temperature (LCST) in aqueous solution [17]. Polymers with LCST behavior undergo significant conformational changes above a certain temperature. These microscopic changes are accompanied by alteration of macroscopic properties, such as solubility. Therefore, LCST polymers, especially poly(*N*-isopropylacrylamide) (PNIPAAm), are used in drug delivery systems or micro system technology [18,19]. Until about 15 years ago the ATRP of *N*-substituted acrylamides seemed to be impossible because of the strong affinity of the acrylamide monomer to complex the copper halide [20]. Masci et al. were the first to present an ATRP system based on the strong binding polydentate ligand Me_6_Tren that can circumvent unwanted competitive complexation of the catalyst [21]. ATRP of NIPAAm using ethyl-2-chloropropionate as initiator and CuCl/Me_6_Tren as catalyst complex in a solvent mixture of water/*N*,*N*-dimethylformamide (DMF) (*v*/*v* 1:1) showed good control over the polymerization process. Moreover, block copolymers of *N*,*N*-dimethylacrylamide (DMAAm) and NIPAAm could be obtained by sequential monomer addition. The work of Masci et al. represents a starting point for ATRP of acrylamides. The CuCl/Me_6_Tren system was successfully used in different solvents, namely dimethylsulfoxide (DMSO), pure water, as well as different alcohols [22,23,24]. Because of the rapid chain growth, especially in aqueous systems, different amounts of the deactivator CuCl_2_ are needed to gain control over the polymerization by shifting the ATRP equilibrium towards the dormant species. Interestingly, the first-order kinetic plots were reported to be linear in the case of water/DMF (*v*/*v* 1:1) but showed significant curvature for ATRP of NIPAAm in DMSO, ethanol, 2-propanol, and *tert*-butyl alcohol [22,24]. This deviation from a first-order kinetic was attributed to a partial oxidation of the copper(I)-complex, especially at the beginning of the reaction. Theoretical explanations for this persistent radical effect (PRE) were reviewed in detail by Fischer [25]. Nevertheless, overall low polydispersities indicate that the polymerization proceeds in a controlled manner. The rapid chain growth in aqueous ATRP was attributed to a high activation rate constant resulting from a disproportionation of copper (I) species yielding copper (0) and copper (II). Meanwhile, copper (II) is known to act as a deactivator; there are contrary positions on the role of copper (0). Percec et al. proposed the highly active copper (0) species to directly activate the dormant species by an outer-sphere single electron transfer, thus leading to living radical polymerization by single electron transfer (SET-LRP) [26]. Recently, Alsubaie et al. reported the synthesis of acrylamide multi-block copolymers via aqueous SET-LRP [27]. On the contrary, Matyjaszewski et al. considered copper (0) to act as reducing agent for the deactivator copper (II), thus leading to an activator regenerated by electron transfer (ARGET)-ATRP mechanism [28]. Despite which ATRP mechanism is applicable, almost all polymer chains should carry the alkyl halide end group, thus being still capable of growing upon further monomer addition. However, quantitative modification of the ω-chain end is reported to be quite difficult, especially for higher polymerization degrees [29]. Consequently, the PNIPAAm chains obtained by ATRP are mostly converted directly in the reaction mixture by sequential monomer addition to yield block copolymers [21,29,30]. But there are only very rare reports on PNIPAAm prepared by ATRP, isolated and characterized, and subsequently used as macroinitiator for block copolymer synthesis [31]. Notably, there needs to be a significant loss of end groups during the purification step. Recently, theoretical investigations of end group stability in the ATRP reaction mixture revealed high end group functionality for moderate NIPAAm conversions [30]. Direct determination of the ω end group is quite difficult due to signal overlapping in the ^1^H-NMR spectra. As an alternative method, mass spectrometry usually requires a purified polymer sample. Furthermore, energy input during the ionization step is known to be crucial for fragmentation of polymer chains but also for fragile end groups [16]. Recently, polyacrylates prepared by single electron transfer-living radical polymerization (SET-LRP) could be proven to possess high end group functionality [32]. Altintas et al. used ARGET-ATRP to prepare poly(styrenes) with bromine ω end groups, which were subsequently converted into terminal alkenes by thermal treatment [33]. Light-induced thiol-ene reactions afforded polymers with various end functionalities. A comparison of the matrix-assisted laser desorption/ionization time-of-flight-mass spectrometry (MALDI-TOF-MS) and ^1^H-NMR spectra of the ARGET-ATRP synthesized polystyrenes revealed the loss of the terminal bromine end groups during MALDI ionization. However, in many cases, the question on whether the loss of end groups occurs during polymerization, purification, or characterization cannot be answered with certainty. With respect to industrial application, changes on the molecular level during polymer fabrication need to be considered as well. Altintas et al. investigated the thermal and thermomechanical stability of end groups and modular ligation points, typically present in ATRP-synthesized polymers [34]. The thermal elimination of bromine ω end groups in ATRP-synthesized poly(styrenes) could be confirmed. Additionally, Altintas et al. could prove that the released HBr catalyzes the cleavage of ester moieties in polymer chains, possibly leading to molecular degradation of the polymer chain.

In this paper, we describe our studies on end group stability of ATRP-synthesized PNIPAAm. The CuCl/Me_6_Tren catalyst complex was used in different solvents (water/DMF, DMSO, acetonitrile) with methyl-2-chloropropionate (MCP) as initiator. The influence of the ATRP solvent as well as the polymer purification process on the end group stability was studied using electrospray ionization (ESI)-TOF mass spectrometry. Conclusions are drawn affecting the synthesis of block copolymers with a PNIPAAm block. Furthermore, a new bromine-based ATRP system is presented, allowing not only the controlled polymerization of NIPAAm but also the block copolymer synthesis of NIPAAm with acrylates and acrylamides. Moreover, different strategies are explained of how to remove residual amounts of unreacted macroinitiator after block copolymer synthesis.

## 2. Materials and Methods

### 2.1. Materials

*N*-Isopropylacrylamide (TCI Europe, >98%, Zwijndrecht, Belgium) was recrystallized from *n*-hexane and dried under vacuum. *N*,*N*-Dimethylacrylamide (Aldrich, 99%, St. Louis, MS, USA) was distilled under reduced pressure and purged with argon. Solketal acrylate was synthesized from acetone, glycerin, and acryloyl chloride according to a literature procedure [35]. Copper(I) chloride (Sigma-Aldrich, 99.999%) was purified by stirring in glacial acetic acid. Copper(I) bromide (Sigma-Aldrich, 99.999%) was recrystallized from aqueous saturated sodium bromide solution. *N*,*N*,*N*′,*N″*,*N″*-Pentamethyldiethylenetriamine (PMDETA) (Aldrich, 99%) was used as received. The ATRP ligand Me_6_Tren was synthesized according to literature [36]. ATRP initiators methyl-2-chloropropionate (Aldrich, 98%) and methyl-2-bromoproionate (Aldrich 98%) were used as received. *N*,*N*-Dimethylformamide (Grüssing, 99.5%, Filsum, Germany), dimethylsulfoxide (Grüssing, 99.5%) and isopropanol (Acros Organics, 99.5% extra dry, Thermo Fisher Scientific, Geel, Belgium) were dried over a 3 Å molecular sieve. Acetonitrile (Fisher Chemicals, >99%, Thermo Fisher Scientific, Geel, Belgium) was distilled and stored over a 3 Å molecular sieve. Bi-distilled water was used for ATRP experiments. For removal of the copper catalyst complex, neutral alumina (Merck, activity level 1, Darmstadt, Germany) was used. All other chemicals were used as received.

### 2.2. Characterization

^1^H-NMR spectra were recorded using a Bruker AVANCE 500 spectrometer (Bruker, Billerica, MA, USA). The solvent peak was used as internal standard. The chemical shift for CDCl_3_ was set to 7.26 ppm and for DMSO-d_6_ to 2.56 ppm. Spectra recording and data evaluation were done using Bruker TOPSPIN 2.1 PL5 software. The poly(*N*-isopropylacrylamide) homopolymers as well as the poly(*N*-isopropylacrylamide-*b*-*N*,*N*-dimethylacrylamide) block copolymers were dissolved in CDCl_3_. For all of the other block copolymers, as well as aliquots withdrawn from the reaction mixture, DMSO-d_6_ was used as NMR solvent. ESI spectra were recorded with the SYNAPT™ G2 HDMS™ from Waters^®^ (Waters Corporation, Milford, MA, USA). Samples for ESI-measurements were composed as follows: 0.5 vol % of the respective PNIPAAm homopolymer in THF (c = 2.0 g/L), 0.5 vol % of NaI in methanol (c = 0.1 g/L), and 99.0 vol % methanol. The system was operated in the positive ionization electrospray mode with a maximal resolution of 20 kDa. Typical parameters for ESI-IMS-TOF measurements read as follows: capillary voltage, 3 kV; sampling cone, 90 V; extraction cone, 4 V; source temperature, 120 °C; desolvation temperature, 300 °C; IMS wave velocity, 700 m/s; and IMS wave height, 40 V. Size exclusion chromatography (SEC) measurements were performed on a modular SEC system consisting of a Knauer Smartline degasser module (Knauer, Berlin, Germany), a Merck-Hitachi L-6000 pump, a Knauer injection valve with a 100 µL sample loop, a Shimadzu CTO-6A column oven (Shimadzu, Kyoto, Japan), and a Waters 2410 RI detector (Waters Corporation, Milford, MA, USA). The system was operated with *N*,*N*-dimethylacetamide (DMAc) as the eluent. Separation according to the hydrodynamic volume of the macromolecules was achieved with two GRAM columns (PSS, 100 and 1000 Å) tempered at 50 °C. Calculation of the molecular mass distribution was based on calibration with narrowly distributed poly(methyl methacrylate) reference polymers (Polymer Standards Service, PSS, Mainz, Germany). Sample concentration was about 3.0 g/L.

### 2.3. AtomTransfer Radical Polymerization (ATRP) of N-isopropylacrylamide (NIPAAm)

The ATRP of NIPAAm was conducted using a copper(I) catalyst in combination with the Me_6_Tren ligand in different solvents. A typical experimental procedure reads as follows: The solvents for ATRP were purged with argon for 15 min prior to use. A dry Schlenk tube equipped with a stirring bar and closed with a septum was evacuated and flushed with argon. A total of 8.7 mg (0.09 mmol) copper(I) chloride, 496.2 mg (4.40 mmol) NIPAAm, and 15 µL Me_6_Tren was added to the Schlenk tube. After evacuating the Schlenk tube and flushing with argon, 1.75 mL of the respective solvent was added. The reaction mixture was degassed by 3 freeze–pump–thaw cycles. After flushing with argon, the Schlenk tube was placed in an oil bath and tempered at 25 °C. Polymerization was started by injecting 10 µL (0.09 mmol) of methyl-2-chloropropionate into the reaction mixture. For polymerization kinetic studies, aliquots of 0.1 mL were withdrawn from the reaction mixture after certain time frames. These samples were analyzed by ^1^H-NMR spectroscopy and SEC according to monomer conversion and molecular mass distribution. Polymerization was stopped either by completely freezing the reaction mixture in liquid nitrogen and exposing it to oxygen or by adding copper (II) chloride solution (24 mg in 1.5 mL solvent). Solvents were evaporated under reduced pressure. The crude polymer was dissolved in THF or chloroform and filtrated over a short column filled with neutral alumina to remove the copper catalyst complex. After evaporating the solvents, the polymer was purified by twofold precipitation in cold diethyl ether (about −50 °C) and onetime precipitation in *n*-hexane at room temperature. Finally, the polymer was dried under vacuum to be obtained as white powder.

### 2.4. Blocking Experiments via Sequential Monomer Addition

Sequential monomer addition was used for blocking experiments to prove the existence of active polymer end groups in the reaction mixture and to synthesize block copolymers. A typical experimental procedure for the synthesis of poly(*N*-isopropylacrylamide-*b*-*N*,*N*-dimethylacrylamide) block copolymers reads as follows: A dry Schlenk tube equipped with a stirring bar and closed with a septum was evacuated and flushed with argon. A total of 27 mg (0.19 mmol) copper(I) bromide, 51 µL (0.19 mmol) Me_6_Tren, and 1.07 g (9.50 mmol) NIPAAm was added. After evacuating the Schlenk tube and flushing with argon, 5 mL of isopropanol and 1 mL of water (both previously purged with argon for 15 min) were injected into the Schlenk tube. The reaction mixture was degassed by 3 freeze–pump–thaw cycles. After flushing with argon, the Schlenk tube was tempered at 0 °C. Polymerization was started by injecting 21 µL (0.19 mmol) methyl-2-bromopropionate. In the meantime, a second solution containing 27 mg (0.19 mmol) copper(I) bromide, 51 µL (0.19 mmol) Me_6_Tren, 0.98 mL (9.50 mmol) *N*,*N*-dimethylacrylamide, 2.5 mL isopropanol, and 0.5 mL water was prepared and degassed in the same way as the reaction mixture mentioned above. After 1 h, a 0.2 mL aliquot was withdrawn from the polymerization mixture to determine the monomer conversion and the molecular mass distribution of the 1st block. Afterwards, the prepared solution of *N*,*N*-dimethylacrylamide and the copper(I) catalyst were added directly to the polymerization system. After stirring at 0 °C for 18 h, polymerization was stopped by freezing the reaction mixture with liquid nitrogen and exposing it to atmospheric oxygen. Solvents were evaporated under reduced pressure. The crude polymer was dissolved in chloroform and filtrated over a short column filled with neutral alumina to remove the copper catalyst complex. After evaporating the solvents, the polymer was purified by precipitation in cold diethyl ether (about −50 °C). After vacuum drying, the block copolymer was obtained as a white powder. The absolute block length ratio was determined by ^1^H-NMR spectroscopy. Molecular mass distribution was investigated by SEC.

Removal of PNIPAAm homopolymer traces was achieved by centrifugation of an aqueous block copolymer solution at about 50 °C and subsequent freeze-drying of the aqueous block copolymer solution.

### 2.5. Synthesis of Poly(solketal acrylate) (PSKA) Macroinitiators

A dry Schlenk tube equipped with a stirring bar and closed with a septum was evacuated and flushed with argon. A 20 mg (0.14 mmol) amount of copper(I)-bromide and 29 µL (0.14 mmol) of *N*,*N*,*N*′,*N″*,*N″*-pentamethyldiethylenetriamine (PMDETA) were added. After evacuating the Schlenk tube and flushing with argon, 1.2 mL (7 mmol) solketal acrylate and 2.5 mL diphenylether (both previously purged with argon for 20 min) were injected into the Schlenk tube. The reaction mixture was degassed by 3 freeze–pump–thaw cycles. After flushing with argon, the Schlenk tube was tempered at 90 °C. Polymerization was started by injecting 21 µL (0.14 mmol) ethyl-2-bromoisobutyrate. After stirring at 90 °C for 1 h, polymerization was stopped by diluting the reaction mixture with 10 mL THF and exposing it to atmospheric oxygen. The polymer solution was filtrated over a short column filled with neutral alumina to remove the copper catalyst complex. Solvents were evaporated under reduced pressure. The crude polymer was dissolved in 5 mL THF and precipitated in 200 mL of *n*-hexane at room temperature. The oily polymer was deposited at the bottom of the flask. The *n*-hexane phase was decanted and the polymer was dissolved in diethyl ether. Removing the ether under reduced pressure afforded the poly(solketal acrylate) (PSKA) homopolymer. Purity of the polymer was confirmed via ^1^H-NMR spectroscopy. Molecular mass distribution was investigated using SEC. Presence of the active bromine end groups was revealed by ESI-IMS-TOF mass spectrometry as well as self-blocking experiments.

### 2.6. Synthesis of Poly(solketal acrylate-b-N-isopropylacrylamide) Block Copolymers

The PSKA homopolymers with active bromine end groups were used as macroinitiators in the ATRP of NIPAAm. A typical experimental procedure for the synthesis of poly(solketal acrylate-*b*-*N*-isopropylacrylamide) block copolymers reads as follows: The obtained PSKA homopolymer was dissolved in 4.0 mL isopropanol. Afterwards, 0.8 mL of water was added. The macroinitiator solution was purged with argon for 20 min. A dry Schlenk tube equipped with a stirring bar and closed with a septum was evacuated and flushed with argon. A total of 27 mg (0.19 mmol) copper(I)-bromide, 51 µL (0.19 mmol) Me_6_Tren, and 1.07 g (9.50 mmol) NIPAAm was added. After evacuating the Schlenk tube and flushing with argon, 2.5 mL isopropanol and 0.5 mL water (both previously purged with argon for 20 min) were injected into the Schlenk tube. The reaction mixture was degassed by 3 freeze–pump–thaw cycles. After flushing with argon, the Schlenk tube and the macroinitiator solution were tempered at 0 °C. Polymerization was started by adding the macroinitiator solution to the ATRP reaction mixture. After stirring at 0 °C for 18 h, polymerization was stopped by freezing the reaction mixture with liquid nitrogen and exposing it to atmospheric oxygen. Solvents were evaporated under reduced pressure. The crude polymer was dissolved in chloroform and filtrated over a short column filled with neutral alumina to remove the copper catalyst complex. After evaporating the solvents, the polymer was purified by precipitation in cold diethyl ether (about −50 °C). Vacuum drying afforded the block copolymer. The relative block length ratio was determined by ^1^H-NMR spectroscopy. Molecular mass distribution was investigated by SEC.

### 2.7. Synthesis of Poly(2,3-dihydroxypropyl acrylate-b-N-isopropylacrylamide) Block Copolymers

The poly (2,3-dihydroxypropyl acrylate-*b*-*N*-isopropylacrylamide) block copolymers (PDHPA-*b*-PNIPAAm) were obtained by hydrolysis of the PSKA acetal moieties under acidic conditions. The experimental procedure was described by Kipping et al [8]. Complete hydrolysis of the acetal moieties was confirmed by ^1^H-NMR spectroscopy. Residual traces of poly (2,3-dihydroxypropyl acrylate) homopolymers resulting from former PSKA homopolymer traces were separated by dialysis of the crude PDHPA-*b*-PNIPAAm block copolymer solution at 50 °C using a Spectra/Por^®^ 6 dialysis membrane (MWCO 1000). Subsequent freeze-drying afforded the purified PDHPA-*b*-PNIPAAm block copolymer. Molecular mass distribution was investigated using SEC.

## 3. Discussion

### 3.1. End Group Stability

The ATRP of NIPAAm using the CuCl/Me_6_Tren catalyst complex was carried out in different solvents. Methyl-2-chloropropionate (MCP) was chosen as initiator. In DMSO a small amount of the deactivator CuCl_2_ was added to increase monomer conversion. The molar ratio between monomer (M) and initiator (I) was kept constant at 50:1 (Table 1). The reaction mixture was tempered at 25 °C.

There are different options of how to stop an ATRP. Usually the reaction mixture is completely frozen in liquid nitrogen and exposed to atmospheric oxygen while thawing. Alternatively, the deactivator CuCl_2_ is directly added to the reaction mixture in excess to stop polymerization. After the polymerization process is aborted, the catalyst complex needs to be removed in order to minimize transition metal contamination of the polymeric product. Normally, passing the diluted reaction mixture through a short column filled with neutral alumina leads to adsorption of catalyst complex. After evaporating residual solvents of the eluate, the polymer can be obtained by precipitation. Dialysis with a fine-meshed membrane can also be utilized to remove the catalyst complex as well as residual amounts of monomer or solvent. Subsequent freeze-drying affords the purified polymer. In this work, ATRP was stopped either by freezing in liquid nitrogen and exposing it to atmospheric oxygen or by adding CuCl_2_ in excess. Afterwards, the solvents of the reaction mixture were evaporated, and the residue was dissolved in either THF or chloroform. The CuCl/Me_6_Tren catalyst complex was removed by adsorption on neutral alumina using THF or chloroform as eluent. After evaporation of the solvent, the polymer was obtained by twofold precipitation in diethyl ether at −50 °C and subsequent precipitation in *n*-hexane at room temperature. In order to exclude the loss of end groups resulting from a poor control over the polymerization at high conversion, polymerization was also stopped after approximately 50% monomer conversion. Table 2 summarizes the termination and work-up strategies.

For each polymerization system (Table 1) four different work-up strategies (Table 2) were tested. After vacuum drying, the purified polymers were characterized using SEC and ^1^H-NMR spectroscopy. Ganachaud et al. investigated the molecular mass characterization of PNIPAAm samples using SEC with THF as eluent [37]. Mainly for higher molecular masses (>10^5^ g/mol) discrepancies between the predicted number-average molecular mass (*M*_n_) and the SEC results were observed. In this work, SEC experiments were performed in a polar organic solvent (*N,N*-dimethylacetamide) with PNIPAAm samples of low molecular mass. Molecular mass distributions were obtained by conventional calibration with narrowly distributed poly(methyl methacrylate) (PMMA) standards, thus obtaining apparent values for *M*_n_ and PDI [38]. Nevertheless, the obtained M_n_ and PDI values could be used to compare the distinct PNIPAAm homopolymer samples. The sample name (Table 3) comprised the polymerization system (capital letter) and the work-up strategy (number). All of the synthesized samples exhibited a narrow molar mass distribution with PDI values lower than 1.20, indicating a steady growth of all polymer chains. The number-average molecular masses ranged between 2500 and 10,000 g/mol depending on monomer conversion. In polar aprotic solvents, such as DMSO and ACN, only a moderate monomer conversion between 38% and 74% could be achieved.

**Figure 1:**
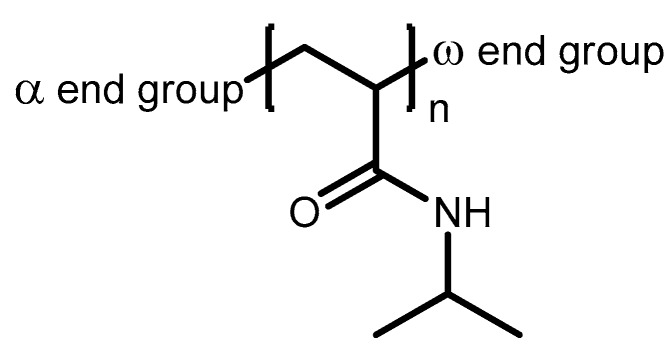
End groups in a PNIPAAm homopolymer chain.

Mass spectrometry was proven to be an efficient method for structural characterization of synthetic polymers, including end group determination [39,40]. Although MALDI-TOF mass spectrometry was successfully used to characterize the end groups of PNIPAAm polymers of different architectures, the difficulty was within sample preparation [41,42]. To obtain a homogeneous mixture of polymer, matrix, and ionizing agent, an extensive process of trial and error had to be conducted. Recently, Brandt et al. attempted to systemize MALDI sample preparation using chemometrics in order to predict suitable matrix and ionizing agents for unknown polymers [43]. Additionally, laser energy as well as sample properties do also have an influence on the MALDI results [44,45]. Although MALDI is known to be a rather soft ionization method, the resulting gas phase ions have a larger internal energy compared to electrospray ionization (ESI) [16]. Thus, this larger internal energy favors spontaneous fragmentation, especially of polymers holding labile end groups. Electrospray ionization mass spectrometry (ESI-MS) offers a considerable alternative, since at least PNIPAAm homopolymers exhibit quite a good solubility in polar solvents (methanol, acetonitrile, cold water) suitable for ESI-MS. A systematic MALDI/ESI mass spectrometry comparison for the characterization of poly(styrene) synthesized by different CRP techniques was reported by Ladavière et al. [46]. Unfortunately, the ESI process usually creates multiply charged polymer chains, thus leading to rather complex mass spectra consisting of several overlapping mass distributions caused by differently charged species. Ion mobility separation (IMS) provides the opportunity to separate ions with respect to their mass, shape, and charge. As a consequence, rather complex ESI mass spectra can be significantly simplified, thus making peak assignment and end group determination easier [47]. All of the synthesized PNIPAAm samples were characterized by ESI-IMS-TOF mass spectrometry as polymer solutions in methanol using sodium iodide as ionizing agent. The analysis of the spectra was mainly focused on determining the molecular mass and the structure of the moieties at both ends of the polymer chain (Figure 1). The α end group was usually represented by the initiating species in the polymerization process, whereas the ω end group depended on how the propagation of the respective chain stopped. For a controlled ATRP, the expected end group at the ω side would be the halogen atom used in the transfer process.

The molar mass of a polymer chain (*M*_i_) depends on the degree of polymerization (*n*_i_), the molar mass of the repeating unit (*M*_RU_), and the molar mass of the end groups (*M*_EG_):

(1) $$M_{i}=n_{i}\cdot M_{\mathrm{RU}}+M_{\mathrm{EG}}$$

During the ionization process in MALDI or ESI mass spectrometry, the polymer chain is charged by attaching a certain number (*z*_i_) of cations with a respective molar mass (*M*_ion_). Thus, the molar mass of the polymer chain increases, but the mass to charge ratio decreases, leading to a shift of the peak signal (*a*_i_) to lower mass-to-charge ratios:

(2) $$a_{i}=\frac{M_{i}}{z_{i}}=\frac{n_{i}\cdot M_{\mathrm{RU}}+M_{\mathrm{EG}}+z_{i}\cdot M_{\mathrm{ion}}}{z_{i}}$$

Unfortunately, the molecular mass of the end group cannot be calculated using Equation (2) because the exact number of repeating units is usually unknown. Therefore, the signals in MALDI or ESI mass spectra have to be evaluated using Equation (3):

(3) $$M_{\mathrm{EG}}\left(y\right)=\;z_{i}\cdot (a_{i}-M_{\mathrm{ion}})+\left(y-x\right)\cdot M_{\mathrm{RU}};$$

$$\mathrm{with}\;x=max\left(b\leq \frac{z_{i}\cdot (a_{i}-M_{\mathrm{ion}})}{M_{\mathrm{RU}}},\;b\;\epsilon \;\mathbb{N}\right)\;\mathrm{and}\;y\;\epsilon \;{\mathbb{N}}_{0};$$

where *x* stands for the highest possible number of repeating units and *y* is a variable natural number. In Equation (3) the molar mass of the end groups is a function of *y*. According to that, different solutions of this function have to be cross-checked with reasonable end group structures resulting from the polymerization process.

**Figure 2:**
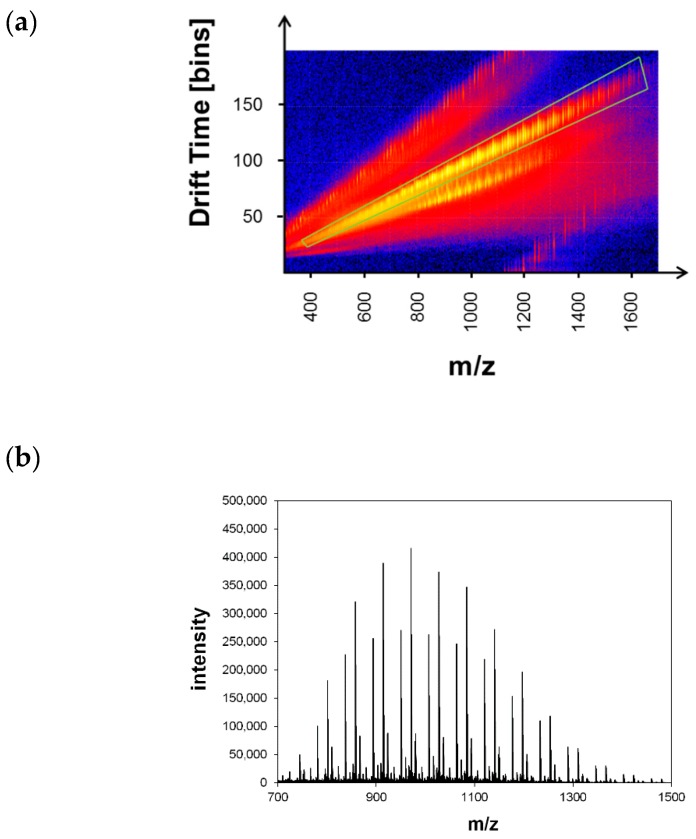

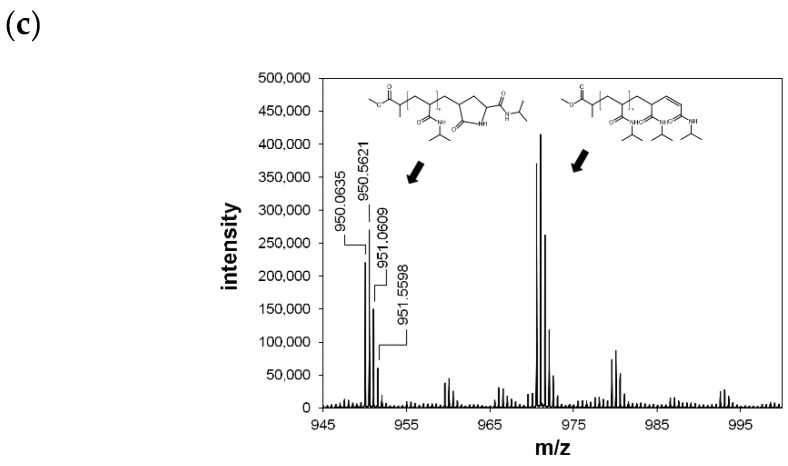
(**a**) Drift time spectrum of the PNIPAAm homopolymer sample C1. The marked region was used for data extraction to obtain a simplified ESI-TOF mass spectra; (**b**) extracted ESI-TOF spectrum; (**c**) magnified region with single series.

Figure 2 shows the ESI-TOF mass spectrum of a PNIPAAm homopolymer. IMS was used to simplify the ESI spectrum by extracting a series of tripe- and quadruple- charged species. A series of high intensity peaks could be found in this spectrum. The isotopic pattern of the high intensity series revealed a difference of 0.50 Da between the single isotopic peaks. For a singly charged species, the distance between two adjacent isotopic peaks should be 1 Da. Thus, the observed high intensity signals must belong to doubly charged molecules. Considering one of these high intensity peaks at an m/z ratio of 950.562 Da, Equation (3) affords 270.904 Da for the molar mass of the end groups (*z*_i_ = 2, *y* = 2, and *M*_ion_ = 22.99 Da). Assuming that the α end group was represented by a 2-(methyl propionyl) group, resulting from the ATRP initiator MCP and the ω end group was based on a pyrrolidinone species, the resulting molar mass would be 270.44 Da, which fitted well with the calculated molar mass.

**Figure 3:**
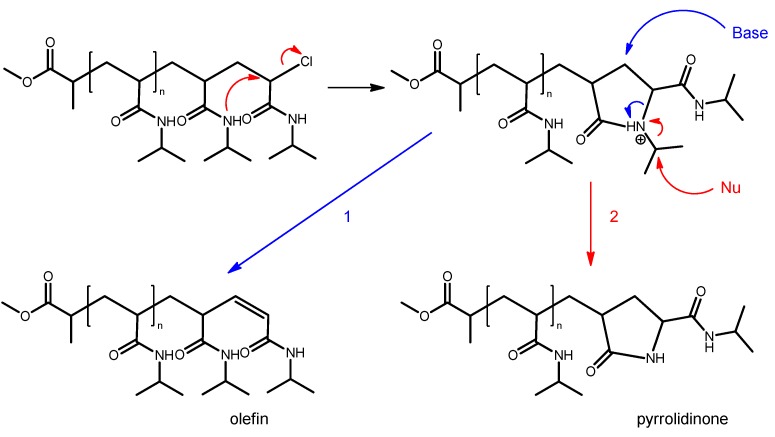
Formation of the unsaturated and pyrrolidinone-based ω chain end group.

This procedure was used to evaluate the ESI-TOF mass spectra of all the synthesized PNIPAAm homopolymer samples listed in Table 3. The results of the end group analysis are summarized in Table 4. All of the polymer chains carried the 2-(methylpropionyl) group (2-MP) originating from the ATRP initiator MCP at the α position. The ω chain end can be usually attributed to an unsaturated chain end (olefin) or a pyrolidinone-based end group. The presence of the unsaturated ω chain ends usually indicates chain termination by disproportionation. In that case, equimolar amounts of chains with a saturated ω chain end should be generated. Since the saturated chain ends were absolutely not observable, the disproportionation theory failed. The publication of Rademacher et al. can be used to derive a new assumption [20]. They suggested that hydroxy groups could be introduced by nucleophilic substitution supported by an intramolecular displacement of the halide group via a cyclic pyrrolidinium intermediate (Figure 3). Instead of replacing the end group, the pyrrolidinium intermediate might also be capable of initiating an elimination reaction in the presence of a base. This would lead to an unsaturated ω chain end. The positively charged nitrogen might also induce a nucleophilic substitution in the isopropyl group, affording the observed pyrrolidinone-based end group. To the best of our knowledge, the presence of the pyrrolidinone-based end group is the first published experimental result that underlines the proposed mechanism of Rademacher et al. for the substitution of the end group via a cyclic pyrrolidinium intermediate in the ATRP of NIPAAm.

The results in Table 3 clearly indicated a controlled polymerization process. The obtained homopolymers exhibited a narrow molecular mass distribution and a direct proportionality between the number-average molecular mass and the monomer conversion. These facts pointed out that there should be a consistent growth of all polymer chains. On the contrary, Table 4 showed the loss of the halide group at the ω chain end, which inevitably led to total inactivity in any further radical chain growth reaction. With respect to that, the question arose: when did the end groups disappear? Although the MALDI and ESI ionization processes were known to cause rather soft fragmentation, the loss of end groups during MS measurements were described in literature [48]. Schilli et al. characterized the end groups of PNIPAAm homopolymers prepared by RAFT polymerization using MALDI-TOF MS analysis. It turned out that most of the polymer chains contained a hydrogen atom or a double bond at the ω position instead of the expected transfer agent end groups. Nevertheless, polymerization kinetics indicated a controlled polymerization process with a consistent growth of all polymer chains. Facing this contradiction, Schilli et al. concluded that the end groups that seemed to originate from radical termination processes were likely the result of fragmentation under MALDI conditions. Since the halide end group can be reductively cleaved, fragmentation during the ESI process seems to be possible as well.

**Figure 4:**
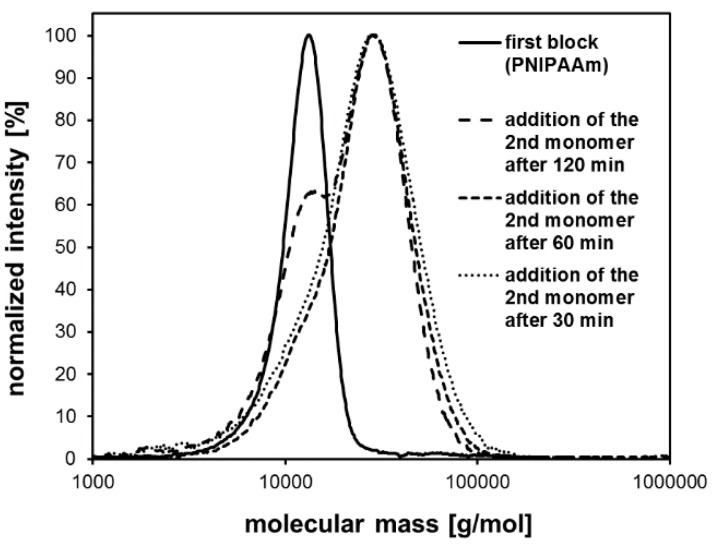
Molecular mass distribution of the PNIPAAm-*b*-PDMAAm copolymers obtained by sequential monomer addition as a result of SEC analysis.

Beside mass spectrometry, blocking experiments can be considered to be an indirect method to prove the presence of active end groups. In the case of the ATRP, only polymer chains possessing the halide end group were capable of growing upon further monomer addition. For the ATRP of NIPAAm in DMF/water (*v*/*v* 1:1), such blocking experiments via sequential monomer addition were carried out. In the first step, NIPAAm was polymerized using the ATRP system A (Table 1). After a certain period of time (30, 60, or 120 min) *N*,*N*-dimethyl acrylamide and fresh copper catalyst complex were added directly to the ATRP reaction mixture. When this copolymerization was finished, the resulting copolymer was purified and analyzed using SEC (Figure 4). 

**Figure 5:**
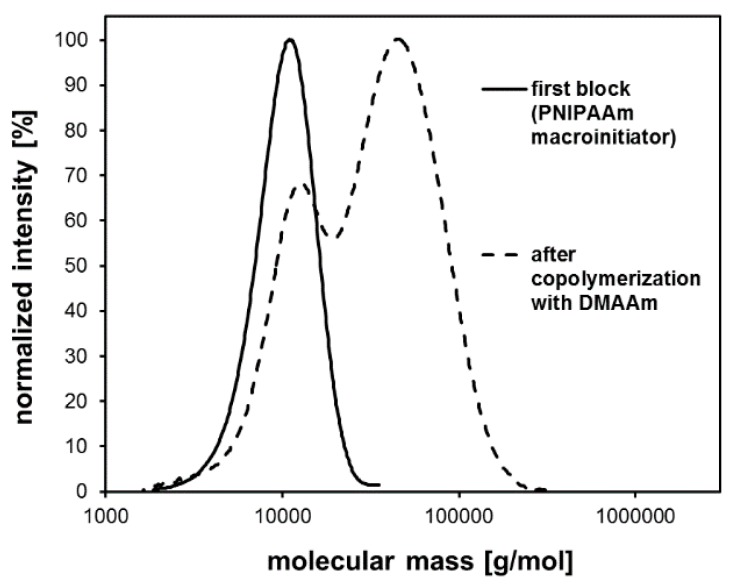
Molecular mass distribution of a PNIPAAm-b-PDMAAm copolymer obtained by polymerizing *N,N-dimethylacrylamide* (DMAAm) using a PNIPAAm macroinitiator.

All of the obtained block copolymers exhibited traces of residual PNIPAAm homopolymer chains that did not grow upon the addition of the second monomer. If the second monomer was added after 30 or 60 min reaction time, the relative number of inactive PNIPAAm chains would be quite low, causing a tailing of the molecular mass distribution towards lower molar masses. If the addition of the second monomer was done after 120 min, the resulting copolymer showed a relatively high number of inactive PNIPAAm chains leading to a significant shoulder in the molecular mass distribution. These results indicated that the halide end groups of the PNIPAAm homopolymers were not stable for more than 1 h in the ATRP reaction mixture. Even below 1 h reaction time a slight loss of end groups could be determined leading to residual traces of unreacted homopolymer chains. Consequently, efficient work-up methods need to be found to remove these homopolymer traces in order to obtain pure block copolymers. Considering this instability of the halide end groups, even in the reaction mixture, it seems not unusual that every attempt to isolate and purify the polymer, even the direct precipitation out of the reaction mixture into diethyl ether, led to a substantial loss of the halide end groups as well (Figure 5). Therefore, the use of acrylamide homopolymers as ATRP macroinitiators did not succeed in our work.

### 3.2. Perspectives for the Synthesis of Diblock Copolymers with a PNIPAAm Block Using ATRP

Block copolymerization offers an elegant method to combine the properties of two chemically diverse homopolymers. Because of their chemical heterogeneity, block copolymers are used as surface and interface active materials [49]. They are able to self-assemble in bulk as well as in solution [50,51]. Block copolymer structures are accessible via living and controlled radical polymerization techniques as well as post-polymerization reactions using click chemistry [52,53,54,55]. There is a wide range of block copolymer applications including nanoparticle synthesis, drug delivery, and organic photovoltaics [56,57,58]. Stimuli-responsive block copolymers are capable of undergoing induced self-assembly triggered by external stimuli [19]. The combination of at least two different stimuli-responsive blocks leads to the actual research field of multi-stimuli responsive polymers [59]. Because PNIPAAm is one of the most intensively investigated temperature-responsive polymers, numerous examples of responsive polymer systems comprising PNIPAAm block copolymers can be found in literature [60,61]. Both ATRP and RAFT are common methods for controlled polymerization of NIPAAm, but still, the use of ATRP was obstructed by the low end group stability of the growing PNIPAAm chain.

Since the stability of the halide end group in the ATRP of NIPAAm is not very high, strategies for block copolymer synthesis are rather limited. Provided that both blocks can be synthesized under the same conditions, sequential monomer addition can be used to obtain diblock copolymers. The reaction time for the first block has to be optimized in order to achieve high monomer conversion (above 95%) as well as to maintain the majority of the halide end groups. Due to radical polymerization, even if it is controlled, there will always be chain termination reactions leading to residual amounts of inactive homopolymer that need to be removed. If both blocks have to be synthesized under different polymerization conditions, the results of the first section clearly point out that PNIPAAm homopolymers prepared by ATRP cannot be used as macroinitiators because the halide end group is easily lost during the work-up procedure. Therefore, the PNIPAAm block will be synthesized in the 2nd place by using a macroinitiator based on the homopolymer that forms the 1st block. Even in that case, removal of homopolymer residues after the block copolymer synthesis is inevitable since not all of the macroinitiator chains are still active and capable of growing. The coupling of two separately synthesized homopolymers and the use of bifunctional initiators complete the synthesis portfolio for diblock copolymers, nevertheless, these methods are not in the scope of this article.

So far, all of the ATRP systems mentioned in Table 1 were based on copper chloride as catalyst and on an alkyl chloride as initiator. However, controlled polymerization of acrylates, for instance, is usually achieved by applying bromine-based ATRP systems. For block copolymerization of acrylates and NIPAAm this discrepancy regarding the halogen in the ATRP system needed to be resolved. Several articles introducing bromine-based ATRP systems for the polymerization of NIPAAm were published [62,63]. Unfortunately, polymerization kinetics were not investigated to prove the controlled character of the polymerization. Experiments using these ATRP systems resulted in PNIPAAm homopolymers with rather broad molecular mass distributions, indicating an uncontrolled polymerization process. Huang et al. established an ATRP system based on a CuBr/Me_6_TREN catalyst complex in isopropanol as solvent [64]. Using a 2-bromoisobutyrate-modified bifunctional initiator block, copolymers consisting of a PNIPAAm and a polypeptide block could be obtained. Decreasing the temperature of the ATRP reaction mixture to 0 °C resulted in PNIPAAm polymers with narrow molecular mass distributions. Nevertheless, monomer conversion only reached about 60%. Although 1st order polymerization kinetics were not observed, low polydispersities and direct proportionality between number-average molecular mass and monomer conversion indicated a consistent growth of all polymer chains [64]. Moreover, Huang et al. successfully applied the CuCl/ Me_6_TREN catalyst complex in combination with the bromine-based bifunctional initiator to obtain narrowly distributed PNIPAAm polymers. However, monomer conversions only reached 70% [64].

It is well-known from literature that the addition of water to an ATRP system often leads an enhancement of reaction rates and monomer conversions [5,65]. Therefore, the addition of water to the ATRP of NIPAAm using the CuBr/Me_6_TREN catalyst complex in isopropanol was investigated. Methyl-2-bromopropionate was used as ATRP initiator.

**Figure 6:**
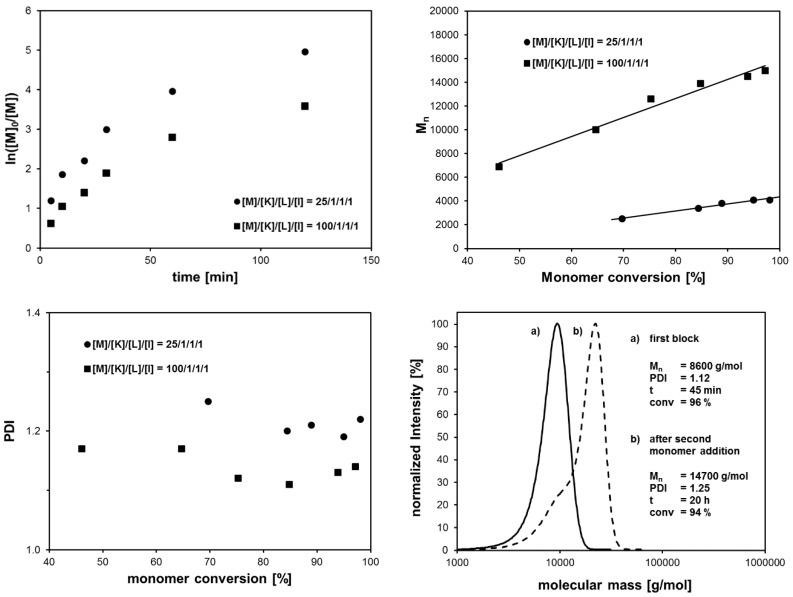
Polymerization kinetics and development of the number-average molecular mass and the polydispersity with increasing monomer conversion and molecular mass distributions (SEC) in the self-blocking experiment.

Table 5 clearly shows that the addition of water to the CuBr/Me_6_TREN catalyst system in isopropanol significantly increased the monomer conversion to over 90%. The molecular mass distribution remained narrow with PDI values lower than 1.2. The reaction temperature needed to be as low as 0 °C. Otherwise, the maximum monomer conversion dropped to 70% and the PDI increased. Figure 6 shows the results of the polymerization kinetics investigation. Monomer conversion was rather fast and exceeded 60% after only 10 min reaction time. In compliance with the results of Huang et al., monomer conversion deviated from a 1st order kinetic. This could be explained by the persistent radical effect (PRE) [25]. Due to rapid chain growth within the first few minutes, the redox equilibrium was still not achieved. Concentrations of growing radicals and the copper (II) species were still changing, thus leading to a non-1st order polymerization kinetic. Nevertheless, all of the polymer chains grew consistently with increasing monomer conversion. A self-blocking experiment revealed that the majority of polymer chains grew further after the second addition of monomer. Still, a small shoulder in the molecular mass distribution indicated that some of the polymer chains suffered from loss of the bromine end groups and were not able to continue chain growth. This issue was not surprising since chain termination reactions were suppressed, but not totally eliminated, in controlled radical polymerization. With respect to block copolymer synthesis, efficient work-up procedures need to be found in order to remove these traces of unreacted homopolymer chains.

**Figure 7:**
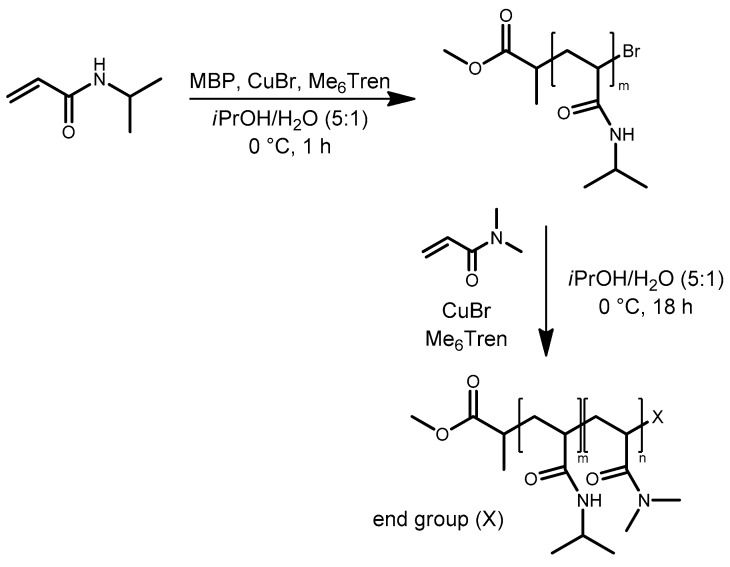
Synthesis of P(NIPAAm-*b*-DMAAm) block copolymers by the sequential monomer addition approach using the bromine-based ATRP system.

Polymerization experiments showed that the introduced bromine-based ATRP system was also suitable for polymerizing *N*,*N*-dimethylacrylamide (DMAAm) to obtain poly(*N*,N-dimethylacrylamide) with a narrow molecular mass distribution. Nevertheless, the achieved monomer conversion was not as high as in the case of NIPAAm. Therefore, block copolymers consisting of a PNIPAAm and a PDMAAm block should be accessible by the sequential monomer addition approach (Figure 7). Indeed, conversion of NIPAAm during the ATRP process exceeded 95% after 1 h polymerization time (Table 6). For shorter PNIPAAm chains synthesized by a monomer-to-initiator ratio of 25, a polymerization time of 30 min was sufficient to convert more than 95% of the initial NIPAAm amount. Addition of DMAAm as second monomer together with fresh copper (II) catalyst complex afforded P(NIPAAm-*b*-DMAAm) block copolymers. The monomer-to-initiator ratio was varied in order to synthesize block copolymers with different absolute block lengths. The conversion of the second monomer amounted to 70% to 85%. As expected, molecular mass distributions of the synthesized block copolymers exhibited a small shoulder, indicating the presence of residual traces of unreacted PNIPAAm chains, thus leading to slightly increased PDI values.

**Figure 8:**
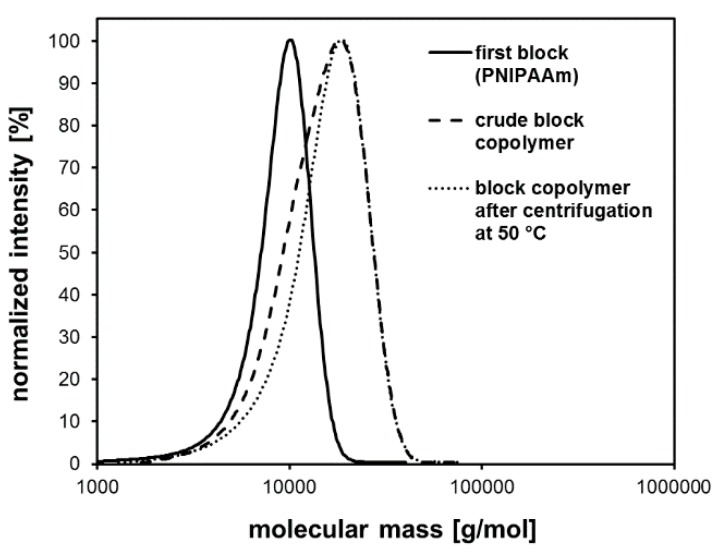
Molecular mass distributions of the first block, the block copolymer before and after centrifugation at 50 °C as a result of SEC analysis (exemplarily for sample BC2).

These traces of PNIPAAm homopolymers could be removed by dissolving the crude block copolymer in cold water and subsequent centrifugation at 50 °C. According to the LCST behavior of PNIPAAm, the block copolymers formed stabilized aggregates and stayed in aqueous solution. Meanwhile, the PNIPAAm homopolymers precipated and could be removed by centrifugation. SEC results clearly confirmed that the shoulder in the molecular mass distribution disappeared (Figure 8). Consequently, PDI values decreased after removing the residual PNIPAAm homopolymer traces (Table 7). 

Using ^1^H-NMR spectroscopy the absolute block copolymer composition could be determined. The absolute block lengths clearly correlated with the chosen monomer-to-initiator ratios during block copolymer synthesis. The slight difference of the number-averaged molar masses taken from NMR and SEC originated from the relative character of SEC molar mass determination and the use of a PMMA calibration curve. From the difference in conversion of NIPAAm before and after addition of the second monomer (Table 7), the number of NIPAAm units (x) that were integrated into the second block could be calculated (Table 7). On average, each PDMAAm block contained significantly less than one NIPAAm unit, confirming the purity of the synthesized block copolymers.

Besides sequential monomer addition, the use of macroinitiators offers a useful alternative for the synthesis of block copolymers. In that case, both blocks can be synthesized under different polymerization conditions. ATRP of acrylates is usually conducted under elevated temperatures using bromine-based ATRP systems. Assuming that the polymerization process proceeds in a controlled fashion, the poly(acrylate) should contain a bromine end group at the ω chain end. Thus, the structure of the poly(acrylate) chain end is similar to the MBP initiator used for the ATRP of NIPAAm. Hence, poly(acrylates) should be suitable macroinitiators for polymerizing NIPAAm with the CuBr/Me_6_TREN catalyst complex, provided that the poly(acrylate) is soluble in the isopropanol/water (*v*/*v* 5:1) mixture.

**Figure 9:**
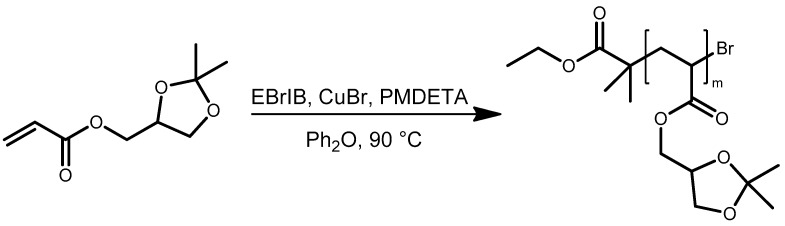
Synthesis of poly (solketal acrylate) (PSKA)macroinitiators using ATRP.

In the current work, solketal acrylate was polymerized in diphenyl ether under ATRP conditions using a CuBr/PMDETA catalyst complex and ethyl-2-bromoisobutyrate (EBrIB) as initiator (Figure 9). Solketal acrylate was chosen since the resulting poly(solketal acrylate) (PSKA) block could be turned into hydrophilic poly(2,3-dihydroxypropyl acrylate) (PDHPA) by hydrolyzing the acetal. The control over the polymerization process was proven by investigation of the polymerization kinetics (Figure S1). The reaction followed a 1st order kinetic and showed a direct proportionality between the number-averaged molar mass and the solketal acrylate conversion. The PDI values were below 1.2. The presence of the active bromine end groups could be confirmed by ESI-TOF mass spectrometry as well as self-blocking experiments (Figures S2 and S3, Table S1). The obtained poly(solketal acrylate) macroinitiator exhibited a number-averaged molar mass of 6100 g/mol and a PDI of 1.16 (Table 8).

**Figure 10:**
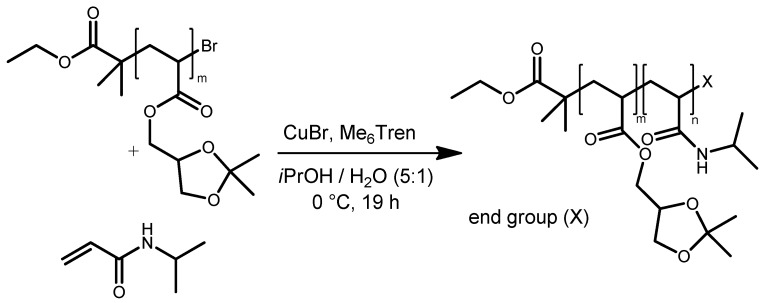
Synthesis of PSKA-*b*-PNIPAAm block copolymers via ATRP with PSKA macroinitiators.

**Figure 11:**
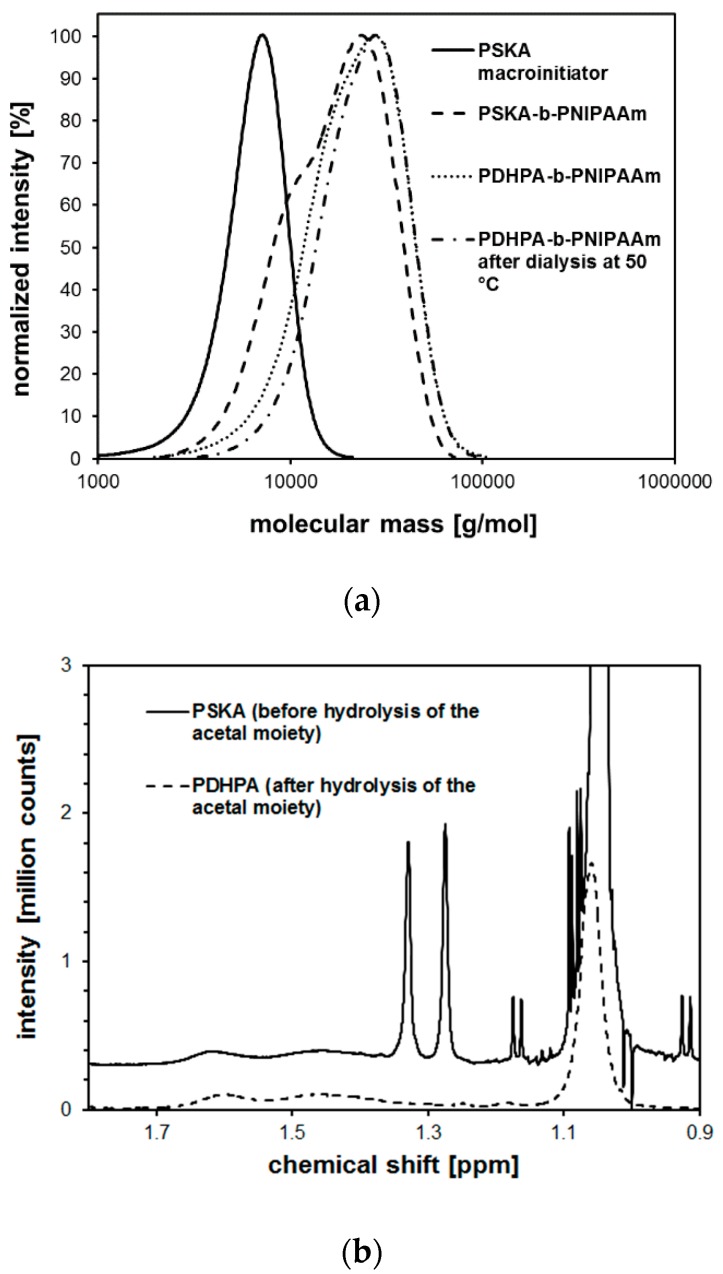
(**a**) Molecular mass distributions of the PSKA macroinitiator, the PSKA-*b*-PNIPAAm block copolymer, and the PDHPA-*b*-PNIPAAm block copolymer before and after dialysis at 50 °C as a result of SEC analysis; (**b**) high field region of ^1^H-NMR spectra of PSKA and PDHPA confirming total acetal cleavage.

In a second step, the PSKA macroinitiator was dissolved in an isopropanol/water (*v*/*v* 5:1) mixture and used for initializing the NIPAAm ATRP with the CuBr/Me6TREN catalyst complex (Figure 10). SEC analysis of the resulting polymer revealed that the molar mass distribution shifted to higher molecular masses (Figure 11). Yet, a shoulder in this distribution indicates the presence of unreacted PSKA homopolymer chains. At this stage, any attempts to remove these traces of PSKA homopolymer did not succeed. Subsequent hydrolysis of the acetal moiety in the PSKA block using glacial acetic acid in THF yielded the hydrophilic PDHPA block. Total cleavage of the acetal was confirmed by ^1^H-NMR, proving the disappearance of the two signals at 1.33 and 1.27 ppm assigned to the two methyl groups in the acetal. Moreover, SEC analysis showed a shift of the MWD to higher molecular masses caused by a higher polarity and better solubility of the PDHPA block in the polar SEC eluent DMAc. However, the MWD still exhibited a shoulder indicating the presence of former PSKA homopolymer traces that were also hydrolyzed to give PDHPA homopolymer.

**Figure 12:**
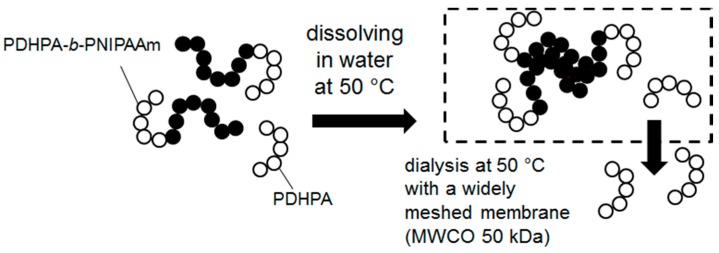
of residual poly(2,3-dihydroxypropyl acrylate (PDHPA) homopolymers by dialysis at 50 °C.

Removing the residual PDHPA homopolymer traces was achieved by dialysis at 50 °C. At this temperature the PNIPAAm block became hydrophobic while the PDHPA remained hydrophilic. Thus, aggregation of the PDHPA-*b*-PNIPAAm block copolymers occurred. Since these aggregates were at least one order of magnitude larger than the single dissolved PDHPA macromolecular chains, they could be separated by dialysis (Figure 12). Subsequent freeze-drying afforded the purified block copolymer with a monomodal and narrow MWD (Figure 11). 

Altering the molar ratio between NIPAAm monomer and PSKA macroinitiator resulted in different block length ratios of the obtained PSKA-*b*-PNIPAAm block copolymers. As expected, hydrolysis of the acetal group in the PSKA block did not affect the block length ratio. The results in Table 9 clearly showed that PDHPA-*b*-PNIPAAm block copolymers with tunable block length ratios and narrow MWDs were accessible via the introduced synthetic route.

## 4. Conclusions

End group stability of PNIPAAm homopolymers prepared by copper-catalyzed ATRP was investigated using ESI-IMS-TOF mass spectrometry and size exclusion chromatography (SEC). The results clearly show a loss of the active halogen group at the ω chain end. Unsaturated end groups as well as pyrrolidinone-based end groups could be determined at the ω chain end. The presence of the pyrrolidinone-based end groups represents the first experimental proof that underlines the loss of halogen end groups via an intramolecular cyclization. Blocking experiments further indicate that even in the reaction mixture, the active halogen end group is only stable for a certain period of time. The slight stability of the halogen end group has a significant influence on the strategy of block copolymer synthesis. Attempts to synthesize and isolate a PNIPAAm macroinitiator failed because there was a major loss of active halogen end groups.

A bromine-based ATRP system for the polymerization of NIPAAm and DMAAm using a CuBr/Me_6_Tren catalyst complex was presented. Kinetic measurements clearly confirmed the controlled character of the polymerization. The introduced ATRP system was used to synthesize block copolymers of NIPAAm and DMAAm with variable absolute block lengths via sequential monomer addition. An efficient work-up procedure based on the LCST-behavior of PNIPAAm was shown to remove residual homopolymer traces. The bromine-based ATRP system also enabled the synthesis of block copolymers of solketal acrylate and NIPAAm. Subsequent hydrolysis of the acetal moiety afforded block copolymers comprised of 2,3-dihydroxypropyl acrylate and NIPAAm. Again, efficient work-up procedures based on the LCST of the PNIPAAm block led to efficient removal of poly(2,3-dihydroxypropyl acrylate) homopolymer traces.

## Figures and Tables

**Table 1:** Chosen atom transfer radical polymerization (ATRP) systems for end group analysis.

System	Solvent	Initiator	[M]/[I]/[Cu^I^]/[Cu^II^]/Me_6_Tren
A	Water/DMF (*v*/*v* 1:1)	MCP	50/1/1/-/1
B	DMSO	MCP	50/1/1.6/0.4/2
C	ACN	MCP	50/1/1/-/1

**Table 2:** Strategies of terminating the polymerization and working up the polymer.

Method	Aspired Monomer Conversion at the Point of Termination	Termination	Solvent for Polymer Work-Up
1	100%	freezing/O_2_	THF
2	100%	freezing/O_2_	chloroform
3	50% ^a^	freezing/O_2_	THF
4	100%	addition of CuCl_2_	THF

^a^ reduced monomer conversion was achieved by reducing the reaction time (see Table 3).

**Table 3:** Characterization of the synthesized poly(*N*-isopropylacrylamide) (PNIPAAm) samples.

Sample ^a^	Reaction Time [h]	Monomer Conversion ^b^ [%]	M_n,SEC_^c^ [g/mol]	PDI ^c^
A1	0.75	91	7910	1.13
A2	0.75	94	8750	1.17
A3	0.08	52	13,490	1.14
A4	0.75	93	8750	1.15
B1	16	70	10,060	1.11
B2	16	69	8870	1.11
B3	6	54	8740	1.11
B4	16	74	9750	1.12
C1	16	38	2670	1.19
C2	16	53	7900	1.13
C3	16	50	8040	1.14
C4	16	44	4190	1.17

^a^ capital letter represents the chosen ATRP system (Table 1); number represents the chosen work-up strategy (Table 2). ^b^ determined via ^1^H-NMR spectroscopy. ^c^ determined via size exclusion chromatography (SEC) with *N*,*N*-dimethylacetamide as eluent and PMMA-standards for calibration.

**Table 4:** Summary of the end group determination using ESI-TOF mass spectrometry.

		END Groups of the PNIPAAm Chain (Main Series)			
	Method	1	2	3	4
System					
**A**		α 2-MP ω_1_ olefin ω_2_ pyrrolidinone	α 2-MP ω pyrrolidinone	α n.a. ^a^ ω n.a. ^a^	α 2-MP ω_1_ olefin ω_2_ pyrrolidinone
**B**		α 2-MP ω olefin	α 2-MP ω_1_ olefin ω_2_ pyrrolidinone	α 2-MP ω_1_ olefin ω_2_ pyrrolidinone	α 2-MP ω_1_ olefin ω_2_ pyrrolidinone
**C**		α 2-MP ω_1_ olefin ω_2_ pyrrolidinone	α 2-MP ω olefin	α 2-MP ω olefin	α 2-MP ω olefin

^a^ end groups could not be determined due to signal overlapping.

**Table 5:** ATRP of NIPAAm using the CuBr/Me_6_TREN catalyst complex and methyl-2-bromopropionate as initiator.

Sample	Solvent	[M]/[C]/[L]/[I]	θ	Time	Conv.	M_n,SEC_^a^	PDI ^a^
			[°C]	[h]	[%]	[g/moL]	
PN17	*i*PrOH/H_2_O (*v*/*v* 5:1)	50/1/1/1	0	19	99	8200	1.11
PN18	*i*PrOH/H_2_O (*v*/*v* 5:1)	100/1/1/1	0	22	91	12,400	1.13
PN19	*i*PrOH/H_2_O (*v*/*v* 5:1)	50/1/1/1	30	21	70	7300	1.20

M = NIPAAm, C = CuBr, L = Me_6_TREN, I = MBP. ^a^ determined via SEC in *N*,*N*-dimethylacetamide using PMMA calibration curve.

**Table 6:** Polymerization conditions for the synthesis of P(NIPAAm-*b*-DMAAm) block copolymers by the sequential monomer addition approach using the bromine-based ATRP system.

Sample	ATRP Reaction Mixture								
	PNIPAAm			PNIPAAm-*b*-PDMAAm					
	[M_1_]/[C]/[L]/[I_1_]	t [h]	Conv. (NIPAAm) ^a^ [%]	[M_2_]/[C]/[L]/[I_2_]	t [h]	Conv. (NIPAAm) ^a^ [%]	Conv. (DMAAm) ^a^ [%]	M_n,GPC_^b^ [g/moL]	PDI ^b^
BC1	50/1/1/1	1	98.2	50/1/1/1	15	98.7	68.3	13,100	1.21
BC2	50/1/1/1	1	98.0	50/2/2/1	18	98.9	82.2	12,900	1.26
BC3	50/1/1/1	1	96.5	100/2/2/1	18	97.7	73.8	16,100	1.39
BC4	25/1/1/1	0.5	95.0	100/2/2/2	18	96.6	71.3	14,900	1.47

M_1_ = NIPAAm, M_2_ = DMAAm, C = CuBr, L = Me_6_TREN, I_1_ = MBP, I_2_ = PNIPAAm; solvent: *i*PrOH/H_2_O (*v*/*v* 5:1); θ = 0 °C. ^a^ determined via ^1^H-NMR spectroscopy. ^b^ determined via SEC in DMAc using PMMA calibration curve.

**Table 7:** Composition, number-averaged molecular mass and polydispersity (PDI) values of the block copolymers after centrifugation.

Sample	ATRP Reaction Mixture		PNIPAAm-*b*-PDMAAm Block Copolymers after Centrifugation			
	Targeted Absolute Block Length Ration	X ^a,b^	m:n ^a^ (NMR, abs.)	M_n,NMR_^a^ [g/mol]	M_n,GPC_^c^ [g/mol]	PDI ^c^
BC1	50:50	0.25	44:47	9700	13,400	1.21
BC2	50:50	0.45	43:54	10,200	13,900	1.24
BC3	50:100	0.60	47:106	15,800	17,600	1.35
BC4	25:100	0.40	24:106	13,200	16,500	1.42

M_1_ = NIPAAm, M_2_ = DMAAm, C = CuBr, L = Me_6_TREN, I_1_ = MBP, I_2_ = PNIPAAm; solvent: *i*PrOH/H_2_O (*v*/*v* 5:1); θ = 0 °C. ^a^ determined via ^1^H-NMR spectroscopy. ^b^ average number of NIPAAm units incorporated into the PDMAAm block. ^c^ determined via SEC in DMAc using PMMA calibration curve.

**Table 8:** Synthesis of the poly(solketal acrylate) macroinitiator.

Sample	PSKA			
	[M]/[C]/[L]/[I]	Conv. ^a^	M_n,SEC_^b^	PDI ^b^
		[%]	[g/moL]	
MI1	50/1/1/1	89	6100	1.16

M = SKA, C = CuBr, L = PMDETA, I = EBrIB; solvent: Ph_2_O; t = 1 h; θ = 90 °C. ^a^ determined via ^1^H-NMR spectroscopy. ^b^ determined via SEC in DMAc using PMMA calibration curve.

**Table 9:** Characterization of the synthesized PDHPA-*b*-PNIPAAm block copolymers.

Sample	m:n ^a^	M_n,SEC_^b^	PDI ^b^
	(rel.)	[g/moL]	
BC5	1.0:2.3	20,700	1.28
BC6	1.0:1.2	23,900	1.24

^a^ relative block length ration determined via ^1^H-NMR spectroscopy; m and n according to Figure 10. ^b^ determined via SEC in DMAc using PMMA calibration curve.

## Supplementary Materials

The following are available online at https://www.mdpi.com/2073-4360/11/4/678/s1, Figure S1: Polymerization kinetics and development of the number-average molecular weight and the polydispersity with increasing monomer conversion for the ATRP of SKA, Figure S2: PSKA self-blocking experiment, Figure S3: (a) Drift time spectrum of the PSKA homopolymer, marked region was used for data extraction to obtain a simplified ESI-TOF mass spectra; (b) extracted ESI-TOF spectrum; (c) magnified region with single series, Table S1: End group assignment of the ESI-TOF peak series.
