# Supplementary material for: End Group Stability of Atom Transfer Radical Polymerization (ATRP)-Synthesized Poly(N-isopropylacrylamide): Perspectives for Diblock Copolymer Synthesis
Source: Polymers (Basel). 2019 Apr 13;11(4):678. doi: 10.3390/polym11040678 (PMC6523552; doi:10.3390/polym11040678)
Supplement: Supplementary file 1 [file polymers-11-00678-s001.pdf]

End Group Stability of ATRP-synthesized Poly(*N*-isopropylacrylamide): Perspectives For Diblock Copolymer Synthesis

Artjom Herberg, Xiaoqian Yu, and Dirk Kuckling[a]

[a] *Universität Paderborn, Department Chemie, Warburger Str. 100, D-33098 Paderborn, Germany*

Supporting Information

Controlled polymerization of solketal acrylate (SKA) using ATRP

The controlled character of the ATRP of SKA using a CuBr/PMDETA catalyst complex was proven by investigation of the polymerization kinetics. Furthermore, ESI-TOF mass spectrometry as well as self-blocking experiments were carried out to prove the presence of active end groups. Experimental procedure was the same as described but the reactant and solvent amounts were doubled. After certain time intervals aliquots of 0.2 mL were withdrawn from the reaction mixture to conduct SEC as well as ^{1}H-NMR measurements.

For self-blocking experiments a PSKA macroinitiator was synthesized as described using a monomer-to-initiator ratio of 50:1. After a reaction time of 60 min the polymerization was stopped diluting the reaction mixture with 10 mL THF and exposure to atmospheric oxygen. The polymer solution was filtrated over a short column filled with neutral alumina to remove the copper catalyst complex. Solvents were evaporated under reduced pressure. The crude polymer was dissolved in 5 mL THF and precipitated in 200 mL of *n*-hexane at room temperature. The oily polymer was deposited at the bottom of the flask. The *n*-hexane phase was decanted and the polymer was dissolved in diethyl ether. 2.5 mL diphenyl ether were added to the polymer solution. The diethyl ether was removed under reduced pressure. The remaining PSKA homopolymer dissolved in diphenyl ether was used as macroinitiator. Apart from that, a new ATRP reaction mixture containing SKA, CuBr, PMDETA and diphenyl ether was prepared as described. The polymerization was started by adding the macroinitiator solution directly to the prepared ATRP reaction mixture. After stirring the polymerization mixture for 18 h at 90 °C, the polymer was isolated as described.

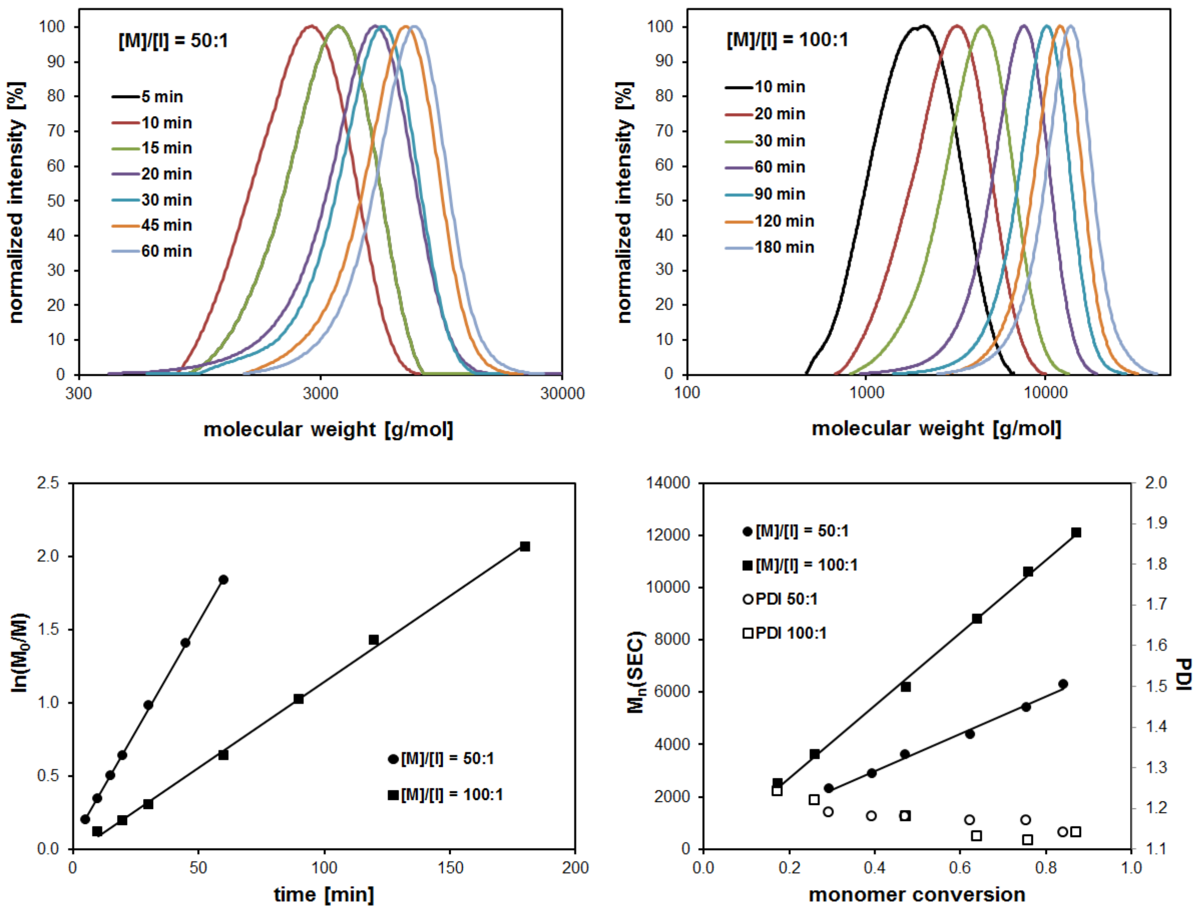

Figure S1: Polymerization kinetics and development of the number average molecular weight and the polydispersity with increasing monomer conversion for the ATRP of SKA

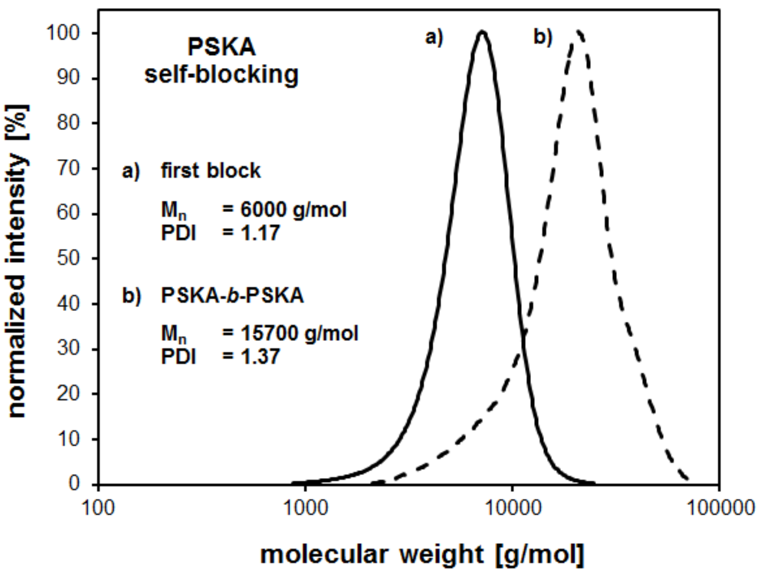

Figure S2: PSKA self-blocking experiment

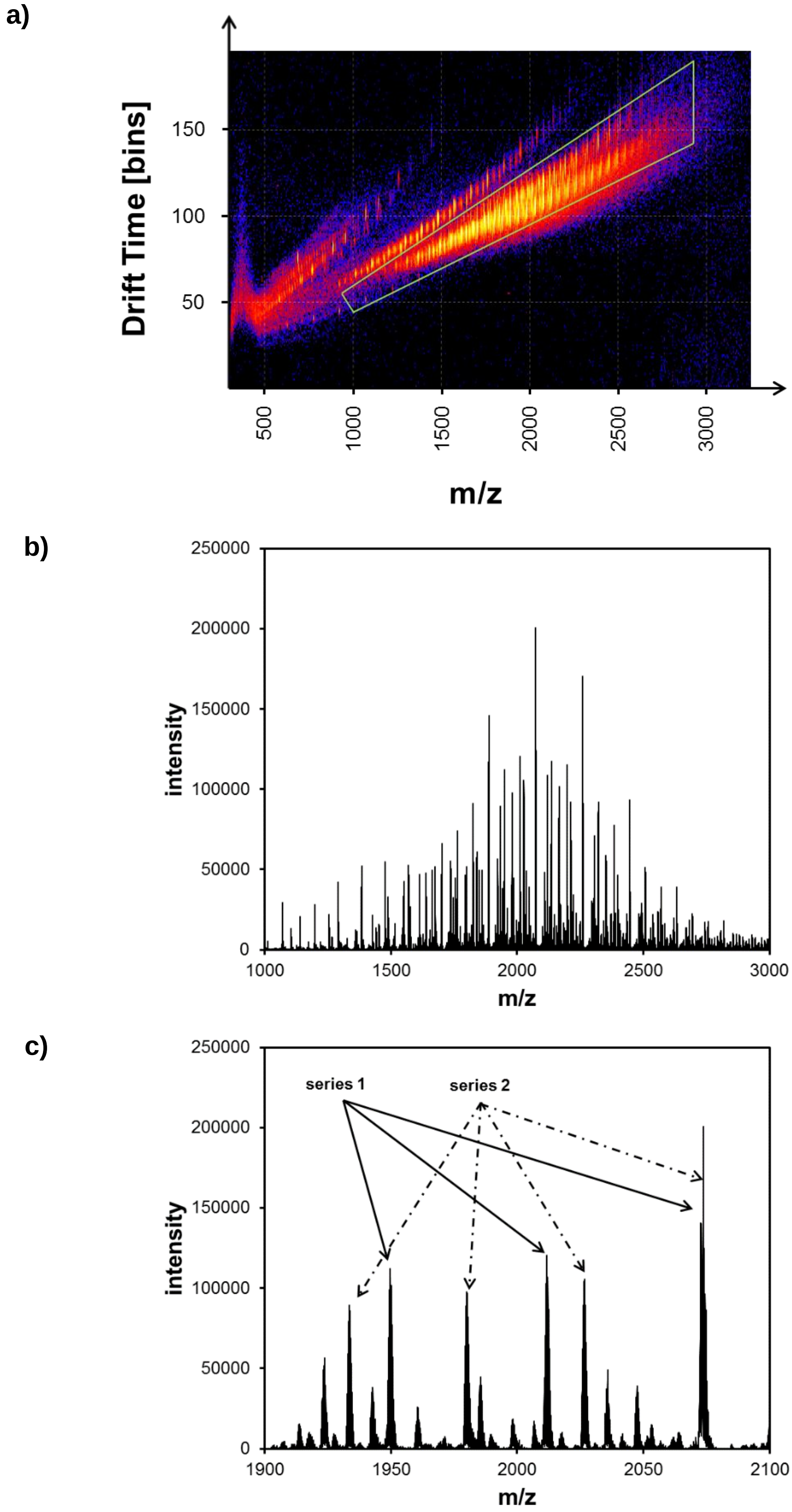

Figure S3: a) Drift time spectrum of the PSKA homopolymer, marked region was used for data extraction to obtain a simplified ESI-TOF mass spectra; **b)** extracted ESI-TOF spectrum; **c)** magnified region with single series

Table S1: End group assignment of the ESI-TOF peak series

Series	α-End Group	Repeat	ω-End Group	Charge State	Adduct	Adduct Charge	Formula
S1	$C_6H_{11}O_2$	$C_9H_{14}O_4$	Br	3	Na	1	$C_6H_{11}O_2$ $[C_9H_{14}O_4]$n Br + $(Na)_3$
S2	$C_6H_{11}O_2$	$C_9H_{14}O_4$	Br	4	Na	1	$C_6H_{11}O_2$ $[C_9H_{14}O_4]$n Br + $(Na)_4$

Figure S1 shows the results of the investigation of the polymerization kinetics. The monomodal molecular weight distributions (MWD) are shifted to higher molecular weights with increasing polymerization time. The linear plot for the 1st order kinetic referring to monomer concentration indicates an intact redox equilibrium between dormant and active species. The direct proportionality between number average molecular weight and monomer conversion confirms a consistent growth of the polymer chains. The PDI values are decreasing during the polymerization process, finally falling below 1.2. Results of the self-blocking experiment are shown in Figure S2. The shift of the MWD after providing new monomer clearly indicates the presence of active bromine end groups. Nevertheless, the MWD tailing towards lower molecular weights can be attributed to residual traces of unreacted PSKA homopolymer.

Figure S3 illustrates the results of the end group determination via ESI-IMS-TOF mass spectrometry. The mass spectrum simplified by IMS contains several peak series resulting from macromolecular ions carrying different end groups and charges. Table S1 summarizes the assignment of the two main series. Both belong to macromolecular ions possessing active bromine end groups at the ω-chain end. The α-end group is derived from the used ATRP initiator ethyl-2-bromoisobutyrate. Series 1 is assigned to triply charged ions, meanwhile, series 2 results from quadruply charged ions. The unassigned minor series belong to macromolecular ion carrying an unsaturated chain end. With respect to that, minor loss of the active bromine end group might occur during the polymerization, as indicated by the self-blocking experiment. Nevertheless, elimination of the bromine end group can also happen during the MS experiment.
